# A Report on Lac Caninum

**Published:** 1884-03

**Authors:** E. B. Nash

**Affiliations:** Cortland, N. Y.


### A REPORT ON LAC CANINUM.

E. B. Nash, M. D., Cortland, N. Y.

Arthur Deliven, age twenty-five; light complexion, dark hair and eyes; spare. Commenced taking Lac. can.$^{2c}$ (B. & T.), Friday evening, October 26th, 1883. Took it through Saturday once an hour, six pills, No. 30, moistened with B. & T.'s 200th. Felt no effects Saturday; omitted it Sunday afternoon. As yet no effect. Commenced again from morning, when he began to feel painful fullness in back, across region of kidneys, much increased toward night. Lying down at night the pain ceased, also pains in calves of legs, but returned soon after beginning to move Tuesday morning and continued all day. Monday morning began to fell a sore place on left tonsil; feels like a sore boil. In the evening seemed not quite so sore, but the back more painful.

Did not feel the soreness of throat much through Monday night. Tuesday morning when he awoke throat felt as though there were lumps in it like two eggs, and sore all the time, especially when swallowing anything. Cold water seemed to relieve momentarily. Tuesday evening examination reveals both tonsils much swollen and very red—left most, and distinct patches on left tonsil. Pain in left occipital region running up when moving head. Pulse 90. Feels feverish, face flushed; urine is unusually frequent and dark. Sometimes darting pain in region of right kidney. First night got to sleep late; sweat profuse during sleep. Felt feverish all night. Morning feels better every way. Has not taken the pills during the night at all.

Miss Nell, age thirty-two; blue eyes, brown, light hair; bilious temperament. Commenced taking Lac. can$^{2c}$ Tuesday, October 30th. Took No. 30 pills, six once an hour. Felt no effect until Friday, when she noticed that when she rose to walk across the store she was very dizzy. Head felt dizzy all the time, but was greatly aggravated when moving. (Has had similar spells before.) Monday night shortly before retiring throat began to feel raw and sore. Did not sleep well Monday night, and in the morning the throat felt full and sore, and a little the worst on the right side. This condition of throat continued until Wednesday, when it seemed to continue downward, and sensation as of a hand in upper part of the chest or lower part of neck began to be experienced. This tightness was very bad, and soon in afternoon, about 2 P. M., commenced a dry cough which was very severe. Thursday, coughs all day in a dry, hacking cough, with occasional severe paroxysms, very fatiguing, with cold hands and perspiration after coughing. In the evening after a hard spell of coughing raised a little blood-stained mucus, a pinkish stain. Frightened her mother. Dreamed the night before that she had hæmorrhage of the lungs. This was caused by the bad feeling in the lungs. Did not cough during the night. Laughing or talking seems to excite the cough. The sensation which provokes the cough is a soreness, fullness, and a sensation as if something might give way and she would have hæmorrhage, fears it. This same senation extends through to the chest, lower inner angle of left shoulder-blade. Chest feels tired and sore. Coughs almost continually while giving me these symptoms. Nose discharges much thin, watery mucus. Every cough hurts her half way down the sternum; feels as though the wind-pipe were peeled inside. Stopped the proving, but the cough and soreness continued obstinate for several days. It finally subsided gradually under the use of Bell 2c.

